# A Novel Surgical Indication for Scheuermann’s Kyphosis

**DOI:** 10.5435/JAAOSGlobal-D-23-00187

**Published:** 2024-03-05

**Authors:** Jason J. Haselhuhn, Kari Odland, Paul Brian O. Soriano, Kristen E. Jones, David W. Polly

**Affiliations:** From the The Department of Orthopedic Surgery (Dr. Haselhuhn, Dr. Odland, Dr. Soriano, Dr. Jones, and Dr. Polly), and the The Department of Neurosurgery (Dr. Jones and Dr. Polly), University of Minnesota, Minneapolis, MN.

## Abstract

Scheuermann kyphosis can be treated surgically to restore proper sagittal alignment. Thoracic curves >70° are typically indicated for surgical intervention. However, patients who have reached their natural limit of compensatory lumbar hyperlordosis are at risk of accelerated degeneration. This can be determined by comparing lumbar lordosis on standing neutral radiographs and supine extension radiographs. Minimal additional lordosis in extension compared with neutral, abutment of the spinous processes, or greater lumbar lordosis standing than with attempted extension suggest the patient is maximally compensated. We present a case of an adolescent boy with Scheuermann kyphosis who had reached the limit of his hyperlordosis compensation reserve. He subsequently underwent a T4 to L2 posterior spinal fusion with T7 to T11 Ponte Smith-Petersen grade two osteotomies. He tolerated the procedure well with no intraoperative complications or neuromonitoring changes. The patient has continued to do well and progressed to normal activity at 5-month follow-up.

Scheuermann kyphosis (SK) was described by Scheuermann^1^ as disturbances in the vertebral epiphyses with wedge-shaped vertebral bodies and pathology resembling Calvé-Perthes osteochondritis. SK can be primarily thoracic or thoracolumbar based on the apex of the kyphosis.^2^ The incidence of SK is relatively high, with rates up to 7.4%.^2^ It is thought to be multifactorial with a genetic component and is a common cause of structural hyperkyphosis in the pediatric population.^3^

Normative values of thoracic kyphosis (TK) reported in the literature range considerably (10 to 55°).^4-8^ Diagnosis of SK is made using Sorensen criteria: anterior wedging of >5° in >3 adjacent vertebrae and TK > 40° or thoracolumbar kyphosis >30°.^9^ Vertebral end plate irregularities and Schmorl nodes are commonly seen, but not necessarily in atypical SK.^9^

There are several accepted surgical indications for SK. While there is substantial variability in the literature regarding the threshold for surgery in terms of the degree of kyphotic deformity (50 to 80°), most agree that curves >70° should be treated surgically.^10-19^ Neurologic deficits, pain refractory to conservative management, cardiopulmonary compromise, and patient dissatisfaction with their appearance are also established indications.

There is often compensatory hyperlordosis of the lumbar spine in patients with SK to maintain sagittal balance. However, once the limit of lordosis is reached, patients cannot compensate for additional progression of their SK, and positive sagittal balance may occur. This has been associated with worse health-related quality of life in adults^20^ and may accelerate degenerative disease in the lumbar spine.^11^ To determine compensatory lordosis reserve, lumbar lordosis (LL) is measured on standing neutral and supine hyperextension radiographs. If they are nearly equal, it signifies that the patient has reached their natural limit of compensation. The senior author has noted that standing LL may actually be higher than LL in attempted supine extension, likely because of a loading effect. This proposed surgical indication has not been described in the literature.

We present an adolescent boy with SK and no remaining compensatory LL reserve who underwent posterior spinal fusion (PSF) with Ponte Smith-Petersen grade two osteotomies (SPO).

## Case Presentation

A 16-year-old boy with SK was referred to spine clinic for evaluation. He denied any symptoms and had no prior treatment, but was discontent with his appearance because of the deformity. Physical examination demonstrated increased TK and LL, with neutral standing sagittal balance.

**Figure 1:**
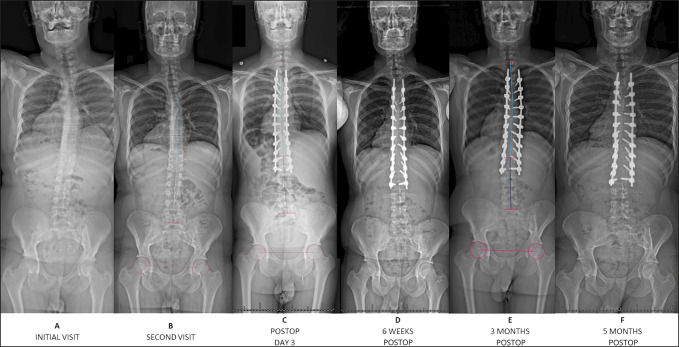
Standing anterior-posterior radiographs from the initial visit to 5 months postoperatively. **A** and **B,** Preoperatively, the patient had severe thoracic kyphosis. **C,** Radiographs show posterior-only multilevel Smith-Peterson osteotomies from T7 to T11 and pedicle screw instrumentation and fusion from T4 to L2. **D**–**F:** Postoperative radiographs obtained at 6 weeks, 3 months, and 5 months.

**Figure 2:**
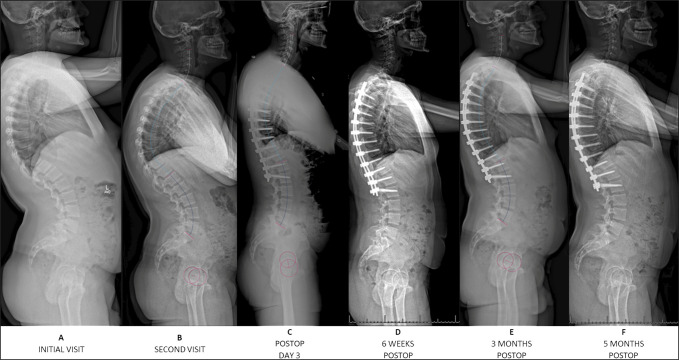
Standing lateral radiographs from the initial visit to 5 months postoperatively. **A** and **B,** Preoperatively, the patient had severe thoracic kyphosis. **C,** Radiographs show posterior-only multilevel Smith-Peterson osteotomies from T7 to T11 and pedicle screw instrumentation and fusion from T4 to L2. **D**–**F:** Postoperative radiographs obtained at 6 weeks, 3 months, and 5 months.

**Figure 3:**
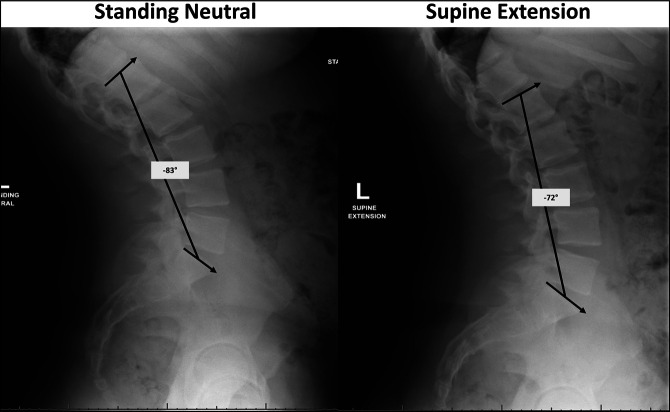
Preoperative lateral lumbar supine extension and standing neutral radiographs demonstrating higher lumbar lordosis when standing versus supine in extension.

Full-spine radiographic measurements included pelvic incidence (PI) 46°, T4 to T12 TK 77°, LL −79°, and T1 to T12 angle 89° (Figures 1 and 2). Lumbar flexion/extension images showed LL was −72° in extension with close approximation of the spinous processes and gapping of the L4 to 5 facet joint due to spinal hyperextension (Figure 3). Lumbar lordosis was measured on a standing neutral lumbar spine image for comparison, which was approximately −83°. This indicated that the patient had reached his limit of LL compensation for the increased TK, and the loading effect of gravity was greater than the forces his spinal extensors could produce. A magnetic resonance imaging (MRI) was ordered to assess for neural compression and/or thoracic disk herniation. It was recommended that he undergo surgical intervention within the next 3 years to prevent additional progression of his disease and lumbar degeneration.

**Figure 4:**
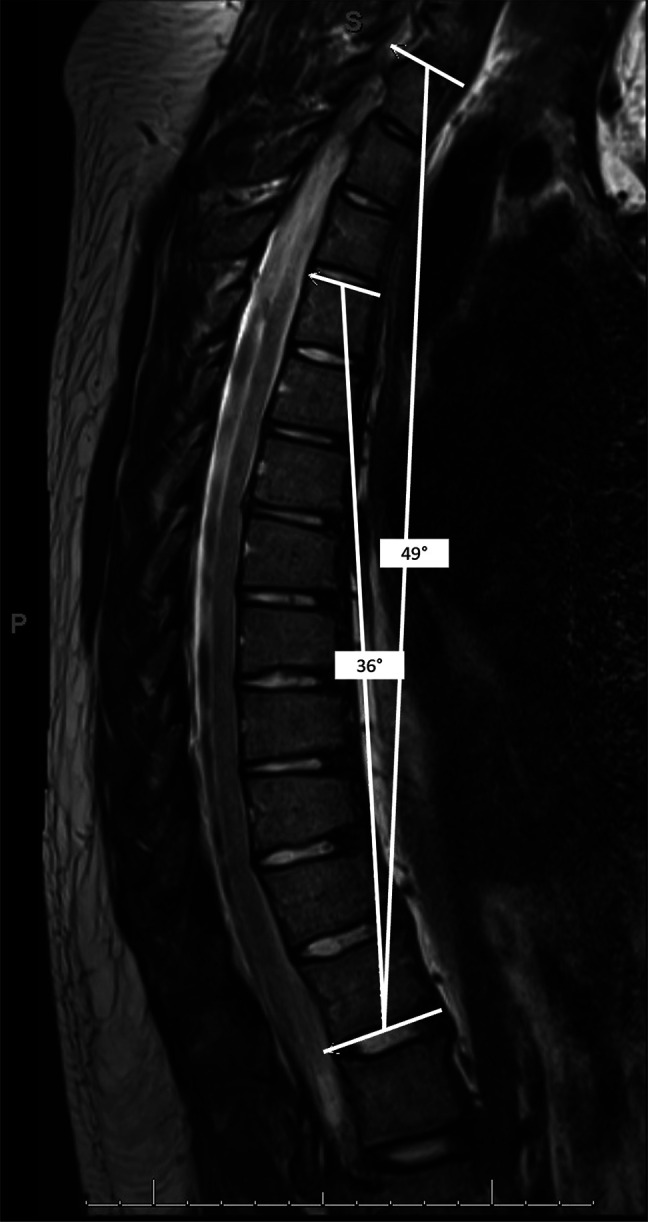
Image showing decreased T1 to T12 angle and T4 to T12 thoracic kyphosis measurements on the T2-weighted thoracic spine magnetic resonance imaging compared with standing measurements demonstrated the flexibility of the patient's curve.

Two months after initial consultation, he returned to the clinic to discuss the MRI findings, which showed effacement of anterior cerebral spinal fluid in the lower thoracic spine and mild anterior wedging of the T7 to T11 vertebral bodies, but no neural compression or disk herniations (Figure 4). His T4 to T12 TK and T1 to T12 angle reduced to 36° and 49° after laying supine for the duration of the examination, respectively, indicating his curve was quite flexible. His Oswestry Disability Index (ODI) was 13.3% and visual analog scale (VAS) was 0. Surgical intervention was offered in the form of PSF and SPO as needed for appropriate sagittal alignment.

**Figure 5:**
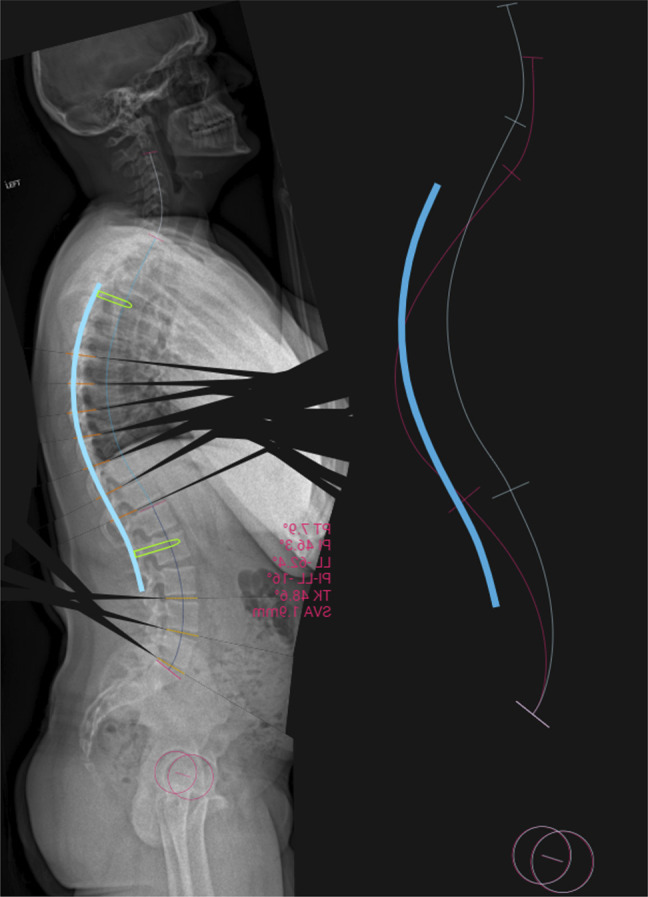
Illustration showing the preoperative surgical plan.

He underwent T4 to L2 PSF with T7 to T11 SPO (Figure 5). Sagittal alignment goals based on preoperative computer modeling (UNiD Adaptive Spine Intelligence, Medtronic) included TK 49°, LL −62°, PI-LL mismatch (PI-LL) −16°, and T1 to T12 angle 55° (Table 1). He tolerated the procedure well with no intraoperative complications or neuromonitoring changes. Radiographs on postoperative day three demonstrated intact instrumentation and improvement of the kyphosis (Figures 1 and 2). Sagittal measurements included TK 51°, LL −52°, PI-LL −6°, and T1 to T12 angle 58°. At the 6-week follow-up, he denied back pain, with an ODI of 0%. Radiographs demonstrated intact instrumentation without evidence of loosening, fracture, or migration.

**Table 1:** Spinopelvic Measurements Based on UNiD Adaptive Spine Intelligence

	Initial Visit	UNiD Sagittal Alignment Goals	Day 3 Postop	3 Month Postop	5 Month Postop
Pelvic tilt, PT (°)	7	8	12	9	9
Pelvic incidence, PI (°)	46	46	46	46	45
Sacral slope, SS (°)	39	38	34	37	36
Lumbar lordosis, LL (°)	−79	−62	−52	−62	−59
PI-LL (°)	−32	−16	−6	−16	−14
T1 pelvic angle, TPA (°)	4	5	11	5	6
Sagittal vertical axis, SVA (mm)	1	2	30	−4	6
T4 to T12 thoracic kyphosis, TK (°)	77	49	51	58	55
T1 to T12 Angle (°)	—	55	58	62	—
Coronal balance (mm)	2	2	16	2	8

At the 3-month follow-up, he again denied back pain, but ODI returned to baseline (13.3%). Full-spine radiographic sagittal parameters included TK 58°, LL −62°, PI-LL −16°, and T1 to T12 angle 62°, which trended toward a 10° correctional loss (Figures 1 and 2). However, at the 5-month follow-up, his ODI decreased to 2.2% and VAS remained 0. Imaging again demonstrated stable instrumentation, and T4 to T12 TK measured 55° and LL −59° (Figures 1 and 2).

## Discussion

This case report introduces a novel surgical indication for SK. The patient had notable SK and lumbar hyperlordosis compensation. Greater LL on his standing neutral compared with supine hyperextension radiographs demonstrated he had exhausted his physiologic compensation reserve for the increased TK. We believe this surgical indication provides prophylactic prevention of accelerated lumbar degeneration of the remaining mobile segments secondary to continual hyperextension if not corrected.

Baastrup disease occurs when there is abnormal approximation of the spinous processes that causes interspinous bursitis, interspinous ligament degeneration, and back pain.^21-26^ This is often attributed to degenerative changes with age, but has also been reported in young patients.^27-29^ The ongoing compensatory lumbar hyperlordosis in this patient resulted in spinous process abutment, facet extension, and disk compressibility, which may predispose patients to developing Baastrup disease and its resulting sequela such as spinal stenosis.^30^ Comparing LL on neutral and hyperextension radiographs is an objective measurement that provides clinicians with data on the patient's compensatory reserve.

Target goals of SK alignment have varied over the years.^7,18,31-33^ The senior author recognizes probable overcorrection in the past, perhaps contributing to proximal junctional kyphosis (PJK) and distal junctional kyphosis issues.^7,8,34,35^ PI-based targets are now discussed, and avoiding large kyphosis-PI mismatch has been shown to decrease the risk of PJK.^18,33,36-38^ Lonner et al^33^ found that an increased postoperative maximum kyphosis to PI ratio was associated with radiographic evidence of PJK (1.5 versus 1.2, *P* = 0.0342). They concluded that patients with low PI require less final kyphosis and patients with high PI require higher final kyphosis. This patient had a preoperative T4 to T12 TK to PI ratio of 1.7, which was reduced to 1.1 in the immediate postoperative period and then increased to 1.2 at the 5-month follow-up due to slight correctional loss with no radiographic signs of PJK. Sarwahi et al^36^ also reported that a large PI to kyphosis mismatch increased risk of PJK and suggested that PJK could be a compensatory mechanism for the spinal imbalance. Nasto et al^38^ found that patients with SK with a high PI-LL mismatch postoperatively were at higher risk of developing PJK. They also found that the magnitude of TK correction correlated with LL reduction and, therefore, concluded that TK correction should be based on PI to prevent large mismatches.

Current alignment targets are often cited as 40 to 50°.^7,35,36^ However, surgical correction of TK < 50° should probably be avoided. The flexibility of the curve is also an important consideration. This patient's T4 to T12 TK decreased to 41° during the supine MRI compared with his initial standing radiographs (36° versus 77°, respectively). Radiographs from postoperative day three demonstrated that 26° of T4 to T12 TK correction was achieved (77° to 51°). This resulted in a T4 to T12 TK-PI mismatch of 5°. However, a 4° correctional loss was noted at the 5-month postoperative visit, which increased the mismatch to 9° (55° to 46°). This loss is similar to early reports by Bradford et al,^15^ but more recent studies have reported minimal correction losses (2.6° to 6°) using a posterior approach.^39^

Combined anterior-posterior spinal fusion for SK has been recommended by many investigators to maximize initial deformity correction, to prevent correctional loss, and to minimize the risk of pseudarthrosis.^14,35,40^ Ponte advocated a posterior-only approach using thoracic pedicle screws, which has been widely used for correction of adolescent idiopathic scoliosis but has not been widely accepted owing to concerns that there would be a risk of correction loss and/or instrumentation failure.^13^ Geck et al^13^ provided additional evidence to support the use of the Ponte procedure with segmental posterior shortening osteotomies, and segmental pedicle screw fixation provides good correction of the deformity in SK. However, recent studies have demonstrated similar results for anterior-posterior and posterior-only approaches.^36,41^

A major motivation for surgical intervention in this patient was self-image. This is supported by his low ODI and VAS. This is in line with Murray et al,^42^ Dambourg et al,^43^ and Ristolainen et al^44^, who all reported minimal functional limitations in patients with curves up to 85°. In addition, Hosman et al reported 19 of 33 patients who considered physical appearance the major motivation for surgery,^7^ which was directly correlated with a low ODI score (7% preoperative vs. 3% postoperative).

## Conclusion

In the adolescent patient with SK who has maximized their lumbar hyperlordosis compensation, surgical correction of the deformity should be considered to prevent accelerated degeneration of the lumbar spine.
